# Etiologies, Gross Appearance, Histopathological Patterns, Prognosis, and Best Treatments for Subtypes of Renal Carcinoma: An Educational Review

**DOI:** 10.7759/cureus.32338

**Published:** 2022-12-09

**Authors:** Ahmed B Mohd, Reem A Ghannam, Omar B Mohd, Rama Elayan, Khaled Albakri, Nesreen Huneiti, Farah Daraghmeh, Eman Al-khatatbeh, Mohammad Al-thnaibat

**Affiliations:** 1 Medicine, Faculty of Medicine, Hashemite University, Zarqa, JOR; 2 Nephrology, Faculty of Medicine, Hashemite University, Zarqa, JOR

**Keywords:** renal cell carcinomas (rccs), hereditary syndromes, renal carcinogenesis, clear cell cancer, renal tumors, renal neoplasm

## Abstract

Of all primary renal neoplasms, 80-85% are renal cell carcinomas (RCCs), which develop in the renal cortex. There are more than 10 histological and molecular subtypes of the disease, the most frequent of which is clear cell RCC, which also causes most cancer-related deaths. Other renal neoplasms, including urothelial carcinoma, Wilms' tumor, and renal sarcoma, each affect a particular age group and have specific gross and histological features. Due to the genetic susceptibility of each of these malignancies, early mutation discovery is necessary for the early detection of a tumor. Furthermore, it is crucial to avoid environmental factors leading to each type. This study provides relatively detailed and essential information regarding each subtype of renal carcinoma.

## Introduction and background

About 3% of solid tumors in adults are renal cell carcinomas, with a slight male predominance [1]. Over the past 20 years, an increase in incidence has been noted, along with claims of a decrease in tumor stage and size in numerous clinical series. Renal cell carcinoma (RCC) incidence differs from country to country. The Czech Republic and North America have the highest incidence rates. Each year, there are about 14,000 mortalities and 63,000 new cases in the United States. It primarily affects males and is more prevalent in urban than rural regions. Sixty years of age marks the pinnacle for incidence rates, which rises with age. Renal cell carcinoma has well-established risk factors such as obesity, hypertension, and smoking [1-3].

The pathogenesis of the malignancy is multifactorial; environmental, behavioral, and genetic associations were linked to the development of the disease [2]. In clinical medicine, managing metastatic renal cell carcinoma remains a significant public health issue. At the time of diagnosis, 30% of cases suffered from metastases, and 50% of patients who have radical nephrectomy for cure subsequently develop either distant metastases or local recurrence [4].

The various renal carcinoma types have different etiologies, gross appearance, histopathological patterns, prognosis, and treatment. This study provides relatively detailed and essential information regarding each subtype of renal carcinoma.

## Review

Renal cell carcinoma

Renal cell carcinomas (RCCs), which develop in the renal cortex, account for 80-85% of all primary kidney tumors [4]. There are more than 10 molecular and histological subtypes of the disease, the most frequent of which is clear cell RCC, which also causes most cancer-related deaths [5].

Patients with RCC present with a palpable abdominal mass, flank pain, and gross hematuria. However, this clinical triad is less common and most patients are diagnosed incidentally. Today, incidental discoveries are the primary source of most diagnoses. This change results from the widespread use of non-invasive radiological procedures for various purposes, such as abdominal CT imaging or ultrasonography [3]. New treatments have been developed due to advancements in the surgical therapy of primary tumors and growing knowledge of genetics and molecular biology of the illness [6].

**Figure 1:**
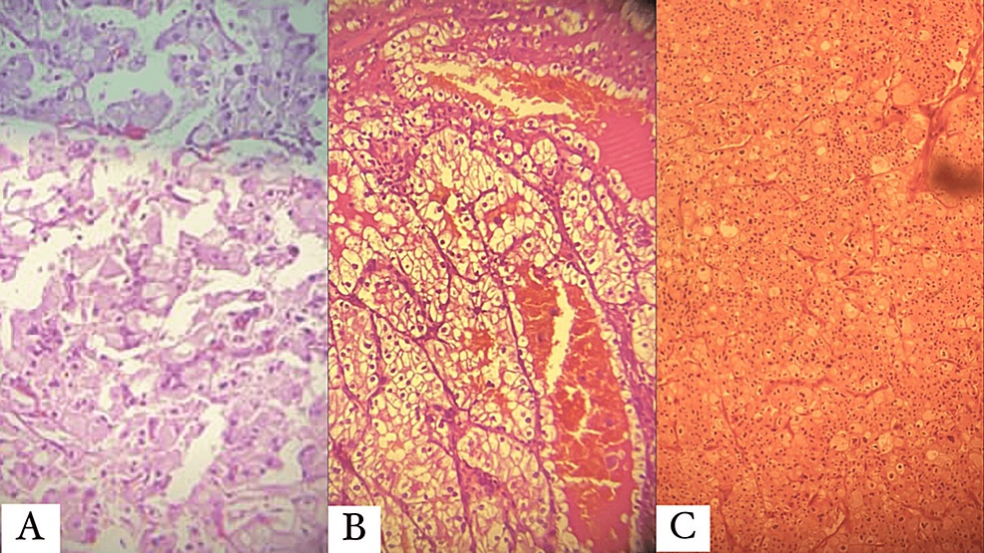
Histological patterns of some subtypes of RCC. The images show (A) clear cell RCC; the neoplastic cells are arranged in sheets with cystic degeneration. Also, focal papillary projections can be seen intermingled by fibro-vascular septae. (B) Clear cell RCC; the neoplastic cells appear with clear cytoplasm and grow in a nesting growth pattern. (C) Chromophobe RCC; the section shows high-grade RCC with sarcomatoid differentiation. RCC: renal cell carcinoma

RCC is divided into several subtypes based on histology. Figure 1 demonstrates histological patterns of some subtypes of RCC. Given the considerable effects of the subtypes on the therapy and prognosis of these neoplasms, the histological classification of RCCs is crucial [7]. These subtypes have different etiologies, gross appearance, histopathological patterns, and immunohistochemistry. We will discuss each subtype and demonstrate the characteristics of each.

Clear Cell Renal Cell Carcinoma

Clear cell renal cell carcinoma (ccRCC) is a deadly neoplasm with a 70% five-year progression-free survival rate and a 24% cancer-specific mortality rate [8]. While many prognostic markers have been studied for these tumors, the morphological characteristics frequently used to forecast patient survival include size, stage, histological tumor grade, tumor necrosis, and vascular invasion [8].

ccRCC can arise from acquired or hereditary factors. Long-term dialysis dependency, diabetes, hypertension, obesity, long-term analgesic usage, and past smoking are some of the common acquired factors. The two most prevalent genetic disorders are the protein polybromo-1 (PBRM-1) gene and the von Hippel-Lindau (VHL) gene. A deletion in the short arm of chromosome 3 is seen in approximately 95% of patients with ccRCC (loss of 3p) [9].

Numerous genes likely connected to renal cancer have been found on the short arm of chromosome 3. The von Hippel-Lindau disease tumor suppressor gene is one of them. The genes PBRM1, RASSF1a, and NRC-1 are also located at 3p [10].

Due to the high concentration of intracytoplasmic lipids, the typical case will appear golden yellow grossly. Less lipid and glycogen are present in higher-grade tumors, which also have a more inconsistent appearance with patches of necrosis and bleeding. Microscopically, approximately half of the cases have either an acinar or solid growth pattern. ccRCC is typically immunoreactive to epithelial membrane antigen, cytokeratin CAM 5.2, PAX-2, and carbonic anhydrase-IX, with diffuse membranous positivity [11]. On imaging, exophytic (outward) expansion, heterogeneity caused by intratumoral necrosis or hemorrhage, and significant uptake of contrast-enhancement agents are typical radiographic findings for ccRCC [4].

The stage of the malignancy, which determines the size and the spread of cancer, must be taken into consideration while selecting the appropriate treatment modality. Localized cancer belongs to stages 1 to 3 depending on its size, while advanced cancer belongs to stage 4. Treatments for patients with ccRCC (i.e., surgery and immunotherapy) depend on how much cancer has progressed. Radical or partial nephrectomy is used for localized cancers, but the physician should also consider the underlying renal function alongside the patient's comorbidities. On the other hand, cryotherapy and radio-frequency ablation are the best treatments for advanced, non-resectable cancers. Furthermore, tyrosine kinase inhibitors are used in metastatic cases [9]. The prognosis is determined by various factors, including where the tumor is located in the body, whether cancer has spread to other body parts, and how much of the tumor was removed during surgery [12].

Papillary Renal Cell Carcinoma

Papillary renal cell carcinoma (PRCC) is a type of renal tumor that develops in kidney tubules, which function as a filtering structure [13]. Papillary tumors resemble long, thin finger-like growths [13]. About 15% of kidney tumors are PRCCs [14]. Histologically, it is marked by tumor cells organized in a papillary fashion within fibrovascular cores [14]. Studies revealed different characteristics between PRCC and ccRCC. For example, PRCCs are characterized by gene duplication on chromosomes 7 and 17; on the other hand, clear cell carcinoma often has 3p deletion [13]. Moreover, the presence of eosinophilic or basophilic cells in tubular architecture served as a histological marker to identify PRCC from ccRCC [14]. Table 1 demonstrates the differences between PRCC and ccRCC.

**Table 1:** The differences between PRCC and ccRCC. PRCC: papillary renal cell carcinoma; ccRCC: clear cell carcinoma

Type of cancer	Papillary renal cell carcinoma	Clear cell carcinoma
Prognosis	Type 1 PRCC: good prognosis, type 2 PRCC: poor prognosis	Variable
Molecular alterations	Type 1 PRCC: a gain of 1 of the chromosomes at 7, 16q, and 17q; type 2 PRCC: lower frequencies of gains of chromosome 17q	3p deletion
Gene mutations	No mutations	VHL, MET, FLCN, TSC1, TSC2, FH, NDUFA4L2, and SDH known as kidney cancer genes

According to a histopathology study based on Fuhrman's nuclear grade and pathologic stage, most tumors were primarily located in the renal poles, well-circumscribed, and averaged 6.7 cm in size. Papillary kidney carcinoma is classified into two types, type 1 and type 2 [13].

Type 1 papillary renal tumor is more likely to develop in people with the genetic condition hereditary papillary renal carcinoma. Moreover, type 1 grows more slowly and spreads less frequently to other body parts than type 2. On the other hand, type 2 papillary renal tumor is more likely to develop in people with renal cell carcinoma and the genetic disease hereditary leiomyomatosis [15]. Therefore, the morphologic classification of type 1 and 2 tumors is hard. However, a study suggested that MUC1 immunostaining may be helpful for classifying these tumors because it is typically positive in type 1 PRCC while nonreactive in type 2 tumors. It is unclear whether immunostaining will offer accurate information for the histologic classification of these tumors [13,15].

**Figure 2:**
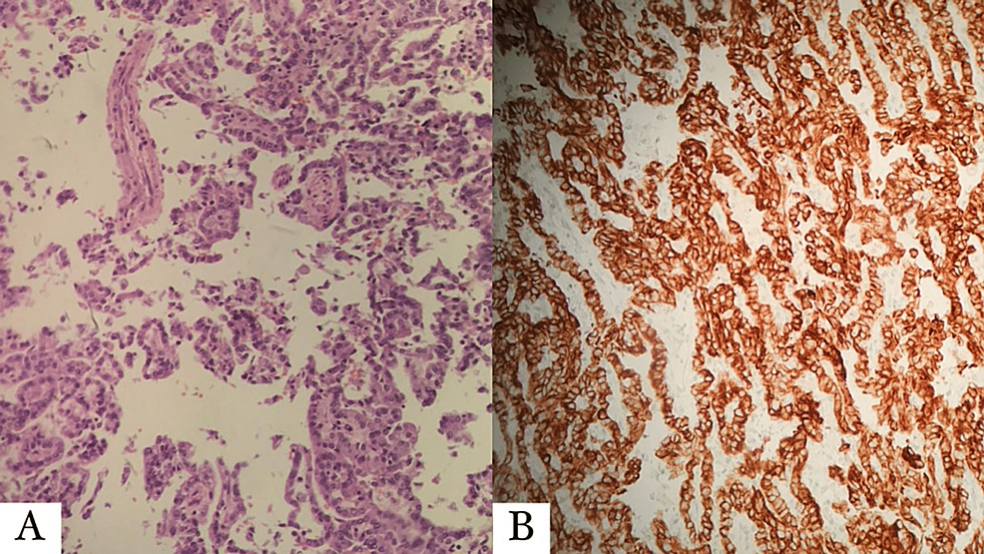
Histological features of PRCC - (A) H&E stain and (B) positive for CK stain. PRCC: papillary renal cell carcinoma; CK: cytokeratins

According to histologic features, type 1 and type 2 PRCC has basophilic and eosinophilic features, respectively. To differentiate between both types, type 1 PRCC has a thin papilla, small round cells containing small oval basophilic nuclei and basophilic cytoplasm; while type 2 has large spherical nuclei, abundant eosinophilic cytoplasm, prominent nuclei with a pseudostratified arrangement, psammoma bodies may be present, and foamy macrophages [16]. Table 2 demonstrates the difference between type 1 and 2 PRCC. Also, Figure 2 demonstrates the histological features of PRCC with different stains.

**Table 2:** The difference between type 1 and 2 PRCC. PRCC: papillary renal cell carcinoma

Type of cancer	Type 1 PRCC	Type 2 PRCC
Histologic features	Thin papilla	Pseudostratified
Nuclei	Small oval basophilic nuclei	Large spherical nuclei, prominent nucleoli
Cytoplasm	Basophilic cytoplasm	Eosinophilic cytoplasm
Psammoma and macrophages	May be present	Present

On the other hand, microscopic studies showed that PRCCs had a papillary or tubular-papillary morphology, presented with foam cells, necrosis, bleeding, and a thick fibrous capsule. Sixty-five percent were high-grade tumors, and 35% were low-grade, with a nuclear grade of III, IV and I, II, respectively [15]. In addition, cytoplasmic characteristics were identified for further differentiation of tumors, such as eosinophilic, basophilic, and mixed, seen in 42%, 34%, and 24% of patients, respectively [15]. As a result, eosinophilic tumors are classified as high-grade while basophilic tumors are low-grade [14,15].

Individuals with hereditary PRCC have a higher chance of getting tumors in both kidneys and a propensity for numerous kidney tumors. This disease is activated by a mutation in germline MET (7q31). As a result, renal cancer risk increases in cases with hereditary PRCC, a rare familial condition [17]. Schmidt et al. discovered germline mutations at 7q31 in six of the seven families with hereditary PRCC. As a result, it altered the MET tyrosine kinase domain, thus constitutive activation. Additionally, 15 different mutations have been found in both sporadic and hereditary PRCC [18].

In hereditary PRCC, trisomy of chromosomes 17 and 7 are also frequently observed and frequently accompanied by MET-activating mutations [5]. However, other pathways are involved in the carcinogenesis of PRCC other than the MET pathway. Such as genetics analysis revealed that trisomy of chromosomes 20, 17, 12, 7, and loss of Y, 9, and 18, are frequently found [17,18]. Schmidt et al. performed an analysis of MET proto-oncogene on 129 cases with sporadic PRCC. The study revealed a limited percentage of mutations (13%). Furthermore, even though no familial history had been mentioned, eight of these mutations were germline. This result shows a reduced frequency of sporadic c-MET mutations. Thus, sporadic tumors rarely contain MET alterations [18].

Thirty-four individuals with papillary renal cancer were the subject of a study by Lubensky et al.; it included both spontaneous and hereditary cases with c-MET mutations, which displayed papillary type 1 histology. Moreover, the expression of the c-MET protein in the membrane and cytoplasm of PRCC tumor cells was further investigated, as a result, a strong MET expression exists [19].

Hereditary leiomyomatosis and renal cell carcinoma, also known as HLRCC, is an autosomal dominant familial syndrome characterized by smooth muscle growth known as leiomyomas on the cutaneous tissue and uterine associated with renal tumors. This disease is a result of a mutation in the fumarate hydratase gene [19]. RCCs associated with HLRCC are aggressive, as 70% of patients die within five years after diagnosis due to metastatic disease. Unilateral and solitary type 2 PRCC is primarily predisposed by HLRCC [20].

The hallmark of HLRCC tumors is the presence of a large nucleus with abundant eosinophilic nucleolus and papillary architecture. All tumors had these characteristics, as did most of the cells within them, whether they lined papillary or tubular structures. In addition, PRCC type 2 has similar morphology, such as prominent nucleoli and peri nucleolar clearance. Among other morphologic characteristics of type 2 PRCC, collecting ducts and cystic development patterns are primary characteristics [20,21]. 

The fumarate hydratase (FH) gene, which is located on chromosome 1q42.3-43, has an autosomal dominantly inherited heterozygous mutation that leads to HLRCC syndrome. A tumor phenotype is developed by inactivating the mutation in the normal FH gene. Moreover, FH gene mutations have been found in 76-93% of families with clinical symptoms associated with HLRCC [21].

Even though metastatic PRCC seems to be less invasive than other RCCs, the prognosis is not better. In a study of 466 patients with metastatic PRCC, the outcome was that individuals with ccRCC had an eight-month longer survival than those with PRCC who had metastatic tumors [22].

To date, no optimal treatment has been approved for PCRR. However, surgery is recommended to improve the survival of patients, along with nephrectomy-targeted therapy. Moreover, PRCC has a better outcome compared to ccRCC. Therefore, tumor removal by radical nephrectomy may be sufficient [22].

Chromophobe Renal Cell Carcinoma

Chromophobe renal cell carcinoma (ChRCC) is a rare RCC subtype, accounting for 5% of all RCC subtypes [23]. This tumor arises from the distal regions of the nephron and is different from the other RCC subtypes histologically and molecularly. ChRCC usually has a slow growth rate; however, metastasis occurs in 5-10% of cases [23].

Compared to ccRCC, the clinical course of ChRCC is less aggressive. The triad of hematuria, flank pain, and palpable mass is rarely seen. At the time of diagnosis, the majority of patients are asymptomatic. Only a tiny percentage of patients present with metastatic disease [24].

As ChRCC is a rare subtype of RCC, its molecular knowledge is limited; however, it differs from other kidney cancers because of its aneuploidy with loss of copies of 1,2,6,10,13, and 17 chromosomes [23]. The exact mechanism of gene dysfunction that leads to aneuploidy is not understood fully [24].

Clear Cell Papillary Renal Cell Carcinoma

Among RCC subtypes, ccpRCC is the fourth most prevalent [25]. Initially, it was known as RCC associated with end-stage kidney disease. However, recent studies showed cases of this neoplasm in patients with healthy kidneys appearing spontaneously [26]. In 2016, clear cell papillary renal cell carcinoma (ccpRCC) was introduced by the World Health Organization classification of renal neoplasia as a novel tumor entity because of its indolent clinical behavior and different morphologic and immunohistochemical features [25].

Patients are usually asymptomatic with mean age of 70 years; some patients may present with flank pain. The tumor has no sex predilection [27]. As the clinical course of this tumor is indolent, it is crucial to diagnose it properly. ccpRCC has different morphologic and immunohistochemical features, but it does overlap with other entities, such as papillary RCC and clear cell RCC. Some of the unique immunohistochemical features of this tumor are the robust diffuse positivity for cytokeratin (CK)7 and cup-like staining for carbonic anhydrase IX (CA9) of its cells [28].

Collecting Duct Carcinoma

Collecting duct carcinoma (CDC) is an epithelial tumor that arises from principal cells of the renal collecting ducts and is considered a sporadic and aggressive tumor that accounts for less than 2% of renal tumors [29]. The diagnostic criteria for this tumor were given by the WHO classification of tumors of the urinary system in 2016 [29].

Ages of CDC patients range from 16 to 90 years at the time of diagnosis, with a 2:1 ratio of more males than females being affected [29]. Clinically, when a patient is diagnosed, they have multiple symptoms with gross hematuria being the most typical one. Other symptoms include back pain, palpable flank mass, and fatigue [29]. As the tumor is highly aggressive, it usually presents as a high-grade tumor with lymph node involvement. Seventy-one percent of patients had metastatic disease with symptoms related to the anatomical site affected [29]. Lack of consistency and specificity of molecular profile for this tumor combined with the aggressive course and bad prognosis makes it hard to evaluate new treatments [30].

Renal Medullary Carcinoma

Renal medullary carcinoma (RMC) is a rare subtype of RCC that accounts for <0.5% of all RCC subtypes and is quite aggressive and sometimes deemed lethal with a <5% survival rate longer than 36 months [31]. The tumor usually affects patients with sickle cell disease or with sickle cell trait, with a higher incidence in males of African or Mediterranean origin at a young age.

Patients with RMC typically present with hematuria and flank pain, and as most patients present with metastatic disease, constitutional symptoms such as fatigue and weight loss can be seen [32]. The presence of sickle hemoglobinopathy can be an indicator for this tumor as well as loss of expression of the tumor suppressor gene INI1 [31].

Unclassified Renal Cancer

According to the WHO classification of RCC, any tumor that does not fit the histological subtypes of RCC would fit in the category of unclassified, which is a rare subtype that accounts for 3-5% of RCC [33]. While the morphologic and genomic overlap between the different subtypes of RCC is increasingly noticed, significant focus is shown toward the rare subtypes and unclassified tumors.

**Figure 3:**
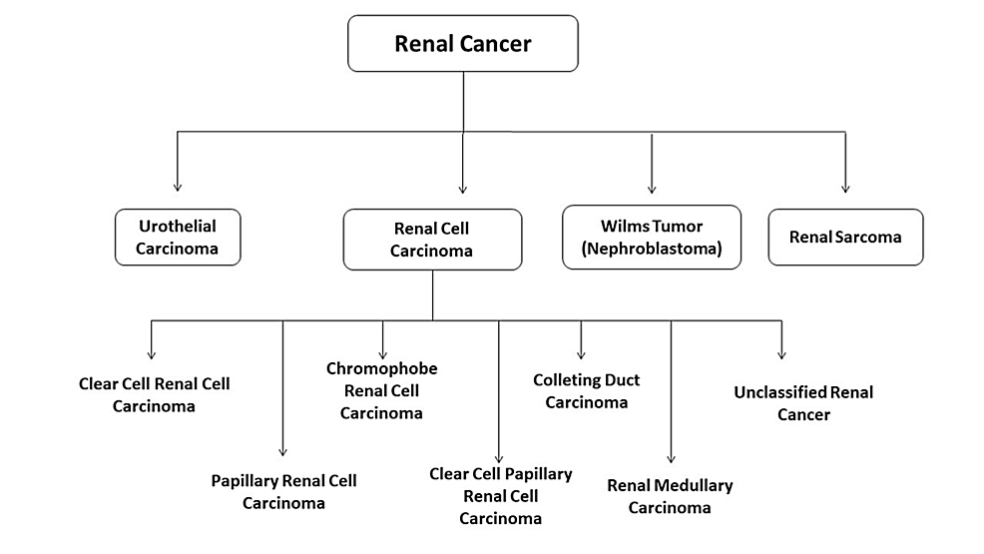
The classification of renal cancer.

The treatment of this type mainly depends on the stage of the tumor and the amenability to resection. For localized disease, surgical resection is preferred, but for metastatic disease and advanced cases, systemic therapy is chosen [34]. Figure 3 demonstrates the classification of renal cancer.

Urothelial carcinoma

Urothelial carcinoma is the fourth most common cancer in males in the United States and the eighth most common cause of death in males [35]. Specifically, a subtype of urothelial carcinoma known as upper tract urothelial carcinoma represents 5-10% of all cases [35]. Upper tract urothelial carcinoma has poor prognosis and is more frequent in males than females with a ratio of 2:1. The major risk factors for this subtype include smoking and dangerous occupational exposure [36]. Major occupational carcinogens include aromatic amines, which can affect people who work in the rubber and chemical industries. Urothelial cancer has also been connected to substances that can harm dyestuff workers such as dianisidine, alpha-naphthylamine, and beta-naphthylamine [36]. Urothelial carcinoma has wide histological diversity, and it has been discovered that many variants are associated with poor prognosis and high-grade risk cancer. So, regarding treatment, immune checkpoint inhibitors (ICI) are more popular now in the oncology field than the standard treatment previously [37].

Gross hematuria is the most common clinical presentation. Due to obstruction by the tumor in one of the kidneys and/or ureter, 25% of patients complain of flank pain [38]. In contrast, 37-80% of patients may reveal hydronephrosis on imaging prior to surgery [38]. It is concluded that hydronephrosis indicates advanced disease, which may affect the decision for chemotherapy and radical resection. A mass in the flank is a rare finding on imaging, and usually, these patients are asymptomatic when a diagnosis is reached. Metastasis is considered when a patient has constitutional symptoms such as weight loss, fatigue, anorexia, and fever [38].

For example, the gross pathology of a subtype, micropapillary urothelial carcinoma, has different features ranging from an ulcerated mass with a gross malignant appearance on examination to another type vividly seen as granular mucosa with low neoplastic potential. The size is also variable, ranging from a few millimeters to large masses. Histologically, micropapillary and sarcomatoid variants were the most common, then comes squamous and glandular variations. The glandular variant is similar to enteric carcinoma and is small tubular or gland-like spaces in typical urothelial carcinoma. The squamous type is identified by clear-cut features of intracellular keratin, intracellular bridges, or keratin pearls. The nested variant is thought of as a malignant type by pathologists. Its patterns vary in superficial and deep portions. The superficial part appears as individual nests with tubules. Their cytological features are usually simple but few can show great cytological atypia. The tumor typically exhibits increased cytologic atypia, an irregular infiltrative pattern, or a clear muscularis propria invasion in the deeper section [39].

Microcystic urothelial carcinoma, a deceptively benign type of urothelial cancer, is characterized by the development of many microcysts, which may result in a false-positive diagnosis of cystitis cystica. A conspicuous, widespread cystic alteration within nests of urothelial carcinoma or urothelial carcinoma with glandular differentiation is the pattern's defining feature. The cysts can range in shape from circular to oval or slit-like, and in size from 1 to 2 millimeters, and contain secretions that could be targetoid occasionally. Most cyst linings are urothelial, with varying glandular morphology interspersed; more giant cysts may also have a flattened epithelium or a denuded lining [40].

It has been demonstrated that E-cadherin is a standalone prognostic marker [41]. Other genes, including APC, RASSF1a, TNFRSF25, EDNRB, and p14, are connected to the development of tumors. IGFBP3 and APAF-1 are separate recurrence indicators. Additionally, the tumor stage and grade are linked with APAF-1. Hypermethylation of DAPK, RAR, E-cadherin, and p16 in urine has been demonstrated to have good sensitivity and specificity for the identification of bladder cancer [41]. According to several studies, measurement of hypermethylation utilizing a panel of tumor-suppressor genes had better outcomes than cytology for the early diagnosis and progression of bladder cancer. Since bladder cancer can be detected using a variety of panels, including RASSF1a/APC/p14, RAR /DAPK/E-cadh/p16, p16/p14/MGMT/GSTP1, and RASSF1a/E-cadh/APC, it is important to consider these panels [41].

Clinical traits, pathogenic variables, and molecular markers are only a few of the potential prognostic factors that have been discovered. The two factors that significantly impact predicting survival in upper tract urothelial cancer (UTUC) patients are lymph node status and tumor stage [42]. Patients who are at risk for non-organ-confined illness can be identified through preoperative assessment for hydronephrosis. A longer time between diagnosis and radical nephroureterectomy is linked to a higher risk of disease recurrence and cancer-specific mortality in the subset of individuals with stage pT2 illness. Lymphovascular invasion, sessile tumor architecture, and extensive tumor necrosis are independent predictors of clinical outcomes for patients with UTUC treated with radical nephroureterectomy [42].

Wilms' tumor (nephroblastoma)

The pediatric age group is mostly affected by Wilms' tumor; many studies registered that one of 10,000 worldwide, in addition to eight cases per million children are less than 15 years of age [43]. With peak incidence between two and five years, it can be found in adults, but it is extremely rare [44]. The tumor is discovered at a later age when it increases in size and causes symptoms, for example, swelling in the abdomen [45].

Some patients present with abdominal pain, hematuria, or hypertension [45]. About 3% of children have one or more relatives with the same tumor; the reason behind that the tumor is associated with congenital anomalies. The National Wilms' Tumor Study Group (NWTS) found that Wilms' tumor is related to genetic alternations that are responsible for normal embryological development, a mutation in the Wilms' tumor 1 (WT1) gene leads to developmental abnormalities in the renal and genitourinary tract, and Wilms' tumorigenesis, causing several syndromes such as Denys-Drash and WAGR syndromes [46]. While on chromosome 11p15, the second Wilms' tumor locus, designated WT2, has been related to Beckwith-Widemann syndrome, characterized by overgrowth in babies, macroglossia, omphalocele, and hyperinsulinism [46].

WT1 is located on chromosome 11p13, which is necessary for normal embryological development of the kidney and gonads. The gene is usually muted in patients with Denys-Drash syndrome, while it is deleted in WAGR syndrome patients [47]. Miller et al. were the first to notice WAGR syndrome (Wilms' tumor, Aniridia - an absence of the iris neither partial nor complete, Genitourinary anomalies, and mental retardation). He studied 440 hospitalized children with Wilms' tumor to find a relationship between the tumor and congenital disability. According to the study's findings, there are various congenital disabilities and mental retardation among the 440 children [48].

Denys-Drash syndrome has a dual clinical picture depending on the gender of the patient. Male patients usually suffered from gonadal dysgenesis, which makes their external genitalia appear either entirely female or opaque with undescended testes. On the other hand, females with this condition may be diagnosed with isolated nephrotic syndrome because they usually have normal genitalia [49].

The treatment of patients depends on the histological class, which is the most significant prognostic factor in this type of tumor. Wilms' tumor is of two types, which are recognized based on the cell types. Classic type, which has no anaplasia, is considered the favorable histology. It accounts for 90% of cases of Wilms' tumor and is associated with a better prognosis. It is also called a triphasic tumor because of the pathological nature of the tumor. It is derived from embryological glomerular structures during renal embryogenesis, and remaining cellular rests (persisting blastemal, dysplastic epithelial tubules, and mesenchymal stromal cells) are considered the component of classic Wilms' tumor [50].

The other unfavorable type is remarked as an anaplastic type. Anaplasia is defined as the presence of morphological changes in a cell (nuclear enlargement, pleomorphism, and hyperchromatic nuclei). This type can be focal or diffuse. Focal anaplasia is anaplastic foci restricted within primary intrarenal tumors and surrounded by non-anaplastic cells. In contrast, when anaplasia is found in multiple regions of the tumor, any extrarenal site, or with metastatic deposits, it is considered a diffuse anaplasia. It is important to note that anaplasia increases the chemo-resistance rates, which leads to poor prognosis [51]. Table 3 demonstrates the difference between the anaplastic and classic types of Wilms' tumor.

**Table 3:** The difference between the anaplastic and classic types of Wilms' tumor.

Type of Wilms' tumor	Anaplastic tumor	Classic tumor
Histology	Unfavorable histology	Favorable histology
Prognosis	Poor prognosis	Good prognosis
Cases affected	10%	90%
Tumor features	Diffuse anaplasia: anaplasia is found in extrarenal sites, Focal anaplasia: anaplasia is limited to intrarenal parts	Derived from the embryological glomerular structure during renal embryogenesis
Component of tumor	Nuclear enlargement, pleomorphism, and hyperchromatic nuclei	Dysplastic epithelial tubules cells, mesenchymal stromal cells, and persisting blastemal cells

NWTS made a study that aims to evaluate the prognosis concerning the histological features; one of the results shows that 4% of patients with focal anaplasia were younger than two years of age and not associated with relapse, while patients with diffuse anaplasia older than two years had a more significant relapse and mortality rates [52].

Collaborative work was done to achieve an excellent outcome in treating children with Wilms' tumor; hence the survival rate increased to 90%. However, before starting with the treatment, staging of the tumor is a crucial step, it enables the cancer care team to decide whether chemotherapy (vincristine, dactinomycin, doxorubicin), surgery, and radiation therapy is the best course of action, so accurate staging at the time of diagnosis is mandatory, the National Wilms' Tumor Study Group (NWTS) has developed a staging system, updated by the Children's Oncology Group Renal Tumor Committee (COG) to characterize the extent of spread of Wilms' tumor based on surgical evaluation before chemotherapy, while the International Society of Pediatric Oncology (SIOP) system recommends using a chemotherapy prior surgery [53].

Both systems developed five stages (1-5), stages 1-4 are categorized as unilateral Wilms' tumors, and stage number 5 as a bilateral tumor. By a risk classification scheme, these protocols give an approach to treating Wilms' tumor patients. Children who are less than two years of age with non-metastatic, favorable histology, stage 1 disease, and both the kidney and tumor weight less than 550 g are recommended for surgery alone. They are considered at low risk for recurrence compared with the risk of chemotherapy which might be higher [54]. Table 4 demonstrates the definition of the various stages of Wilms' tumor and the treatment for each one.

**Table 4:** The definition of the various stages of Wilms' tumor and the treatment for each one.

Stage	Stage definition	Treatment
Stage 1	Tumor is confined to one kidney with an intact capsule	Completely excised. Dactinomycin+vincristine for 18 weeks
Stage 2	Cancer is spread to the kidney and in the fat, soft tissue, or blood vessels near the kidney. It may also spread to the renal sinus	Completely excised. Dactinomycin+vincristine for 18 weeks
Stage 3	The tumor is found in areas near the kidney. Also, the tumor may spread to the abdomen and to nearby lymph nodes	Completely excised. Dactinomycin+vincristine for 18 weeks
Stage 4	Metastasis to lung, liver, bone, and brain	Completely excised from the kidney and surgery may be an option to remove any liver tumors
Stage 5	The tumor is found in both kidneys	Completely excised from both kidneys

The relapse is higher in a patient with WT1 mutation and 11p15 loss of heterozygosity who was diagnosed as stages 1, 2 and treated by nephrectomy. However, in these patients, along with more than two years of age in stage 1 patients, the weight of kidney and tumor was more than 550 g; in addition to the surgery, they require a combination of chemotherapy without radiotherapy, and patients with stage 3 need additional radiation therapy depending on lymph node involvement or peritoneal contamination. Stage 4, after chemotherapy, radiation therapy is required in the lung field if lung metastases do not completely respond or patients with regional lymph node metastases [54,55].

The point of view of SIOP is that giving preoperative chemotherapy will decrease the tumor size [56]. Therefore, administer vincristine and dactinomycin for four weeks to patients with unilateral tumors, while in case of metastasis, triple therapy by adding doxorubicin for six weeks before surgery [53]. For bilateral tumor preoperative, postoperative chemotherapy and resection are advised by both COG and SIOP, with renal parenchymal sparing resection, so the renal function of these kids may be preserved, and kidney transplantation is advised [57].

Renal sarcoma

Renal sarcoma is an uncommon type of renal tumor that initially occurs in the kidney's blood vessels or connective tissue and is caused by abnormal (cancerous) growth of the cells in renal blood vessels or connective tissues. This type accounts for only 1% of all renal tumors [58,59]. Leiomyosarcomas (LMSs) is a type of cancer that is more common in females and typically occurs in the fourth and sixth decades of life. The tumors most commonly occur in the right kidney, liposarcomas make up about 10-15% of all kidney sarcomas, and LMSs are responsible for 50-60% of them [58,59].

Like other types of cancer, renal sarcoma has many risk factors that help us to know a person's probability of getting this type of cancer. Some of them cannot be changed as age and family history, and others are modifiable [60].

Renal sarcoma has many acquired risk factors, such as smoking, age groups between 50 and 70 years, obesity, asbestosis, and hepatitis C. There is a higher risk for sarcoma in black people, compared to white people, and in men, compared to women. Some medications, such as aspirin, ibuprofen, and acetaminophen, are known to increase the risk for this type of cancer. Hereditary risk factors include hereditary leiomyomatosis, renal cell carcinoma (HLRCC), Birt-Hogg Dubé (BHD) syndrome, hereditary papillary renal cell carcinoma (HPRCC), and von Hippel-Lindau (VHL) syndrome [60].

Based on a study, 35 patients presented with colicky pain and an abdominal mass. These two cardinal symptoms may occur alone or combined with other symptoms, as it happened with 31 patients. In addition, gross hematuria was noted in nine patients, and weight loss related to dyspepsia, fatigue, and loss of appetite were discovered in 22 patients. These symptoms' duration varies from patient to patient. Clinical examination findings based on the later study were an abdominal mass with variable sizes, varicocele, a temperature of (101-103°) Fahrenheit, and blood pressure of 140/90 mmHg. However, these findings were not found in all patients. In a study involving patients diagnosed with renal sarcoma, renal sarcoma presented as a palpable lump on the side, or on the lower back and is associated with hematuria, anemia, weakness, hypertension, anorexia, night sweating, and unintentional weight loss [58,60].

Renal sarcoma of the kidney appears as a fleshy cut surface ranging in color from white to pale tan-grey and distorts most nearby healthy kidneys. Some tumors have cut surfaces that range from soft, mucoid to cystic and contain bleeding or necrosis foci. Although the physical border between the tumor and the neighboring healthy kidney is typically clearly defined, a histologic examination may reveal microscopic trapping of native tubules and glomeruli at the tumor's invasive edge. In some instances, there may be obvious signs of renal vein invasion [61].

Histopathology of renal sarcoma can mimic other pediatric renal cancers and has a highly varied histologic appearance, making histologic diagnosis potentially difficult. Although many different histologic patterns have been described, the classic pattern is the most frequently found and is typically present, at least focally, in the majority of cases. Traditionally, plump ovoid cells are grouped in broad trabeculae or nests and divided by regularly spaced and arborizing fibrovascular septa. These septa, which have narrow capillaries or bands of fiber collagen that are abundantly thickened, are frequently described as having a "chicken-wire" appearance [61]. The cord cells have uniform round-oval nuclei with fine chromatin, inconspicuous nucleoli, and transparent cytoplasm. The hypochromic finely distributed chromatin is a significant cytological trait that aids in separating this tumor from imitators. In the extensive study by Argani et al., cells with "Orphan Annie" eye nuclei that seemed empty frequently appeared [61]. Mucopolysaccharide makes up the interstitial matrix, which helps explain why the cells appear transparent. Renal tubule entrapment near the lesion's edge is caused by the subtly expanding cord cells, which is a defining feature [61].

It is well known that renal sarcomas have complex genetic makeups and typically exhibit "chaotic" karyotypes, including aneuploidy and polyploidy. Studies have revealed that the tumor suppressor proteins p16 and p53 are overexpressed in LMS; hence they might be utilized as prognostic indicators. There are genomic mutations that significantly impact the disease prognosis and are associated with a high metastasis potential and an independent prognostic factor for shorter survival times in patients with LMS. There are genomic mutations that significantly impact the disease prognosis and are associated with a high metastasis potential and an independent prognostic factor for shorter survival times in patients with LMS such as deletions in the 4q31 and 18q22 regions and duplication at 1q21 [62].

Diagnosis is made by ultrasound, x-ray, PET, CT, or MRI scan, if a tumor is found, a pathologist will take a kidney biopsy, or a small tissue sample to examine under a microscope. Given the rarity of renal sarcoma, a specialist should be consulted to guarantee a correct diagnosis. The prognosis of renal sarcoma depends on many things, such as surgical, anatomical, and some radiological features, but the most important prognostic factor is surgical resection [60].

The factors associated with disease-specific mortality are the presence of an inoperable tumor or incomplete surgical resection. In addition, a high risk of recurrence is associated with surrounding reactive tissue; that is why resection of the tumor with the reactive tissue is highly recommended. Another prognostic predictor is the presence of metastasis at the time of diagnosis, as patients with metastasis have a short mean survival rate. The most effective therapy in cases of renal sarcoma is the total surgical resection of the tumor [59]. As this article presented, renal cancer types differ in their microscopic, histologic, and gross appearance. Table 5 demonstrates each renal cancer's microscopic, histologic, and gross appearance.

**Table 5:** Each renal cancer's microscopic, histologic, and gross appearance.

Type of cancer		Histologically/microscopically	Grossly
1. Renal cell carcinoma	Clear cell renal cell carcinoma	Usually tightly packed nests and sheets of cells with a unique membrane and cytoplasm. In high-grade tumors or in close proximity to sites of necrosis or bleeding, granular eosinophilic cytoplasm is seen [63].	The typical case will appear golden yellow grossly. Less lipid and glycogen are present in higher-grade tumors, which also have a more inconsistent appearance with patches of necrosis and bleeding [11].
	Papillary renal cell carcinoma	Type 1 PRCC has basophilic features, and type 2 has eosinophilic features. To differentiate between both types, type 1 PRCC has a thin papilla, small round cells containing basophilic cytoplasm, and small oval basophilic nuclei. While type 2 has large spherical nuclei, abundant eosinophilic cytoplasm, prominent nuclei with a pseudostratified arrangement, foamy macrophages, and psammoma bodies may be present [16].	A fibrous pseudocapsule usually encloses the tumor. It typically has a tan or brown appearance, however, when hemorrhages are present, it has a brown appearance. However, when necrosis is present, it has a yellow appearance [64].
	Chromophobe renal cell carcinoma	It consists of nests, sheets, alveoli, or trabeculae with well-defined plant-like cell boundaries (vegetable cells). It is typically a solid growth. There are two types of tumor cells. Type 1 consists of large polygonal cells which have a hard cell border. However, type 2 consists of smaller cells with finely granular eosinophilic cytoplasm [65].	Tan to light brown, well-circumscribed, unencapsulated. Areas of necrosis, bleeding, and small cysts are seen [65].
	Clear cell papillary renal cell carcinoma	The general architecture pattern might be tubular, papillary, solid, cystic, acinar, or a combination of these, but the tubular-papillary pattern with recognizable branching tubules is the most typical architecture. The nuclei frequently have small, round to oval shapes, regular nuclear membranes, and small to barely perceptible nucleoli [66].	Small, frequently enclosed, and with a distinct, thin, fibrous capsule [66].
	Collecting duct carcinoma	Cords or tubular structures which are highly infiltrative structures are embedded in an inflammatory desmoplastic stroma. Large, pleomorphic nuclei with prominent nucleoli and coarse chromatin characterize these cells [67].	The majority of collecting duct carcinomas are large, medulla- and cortex-infiltrating masses. The tumor has invasive borders and is hard, light gray to tan-white on the cut surface. Cysts, necrosis, and hemorrhages are frequent [67].
	Renal medullary carcinoma	Numerous morphologic patterns, including those that are tubulopapillary, tubular, glandular, adenoid cystic-like, microcystic, reticular, and solid patterns that overlap with patterns for collecting duct carcinoma. Pleomorphic tumor cells have eosinophilic cytoplasm, hyperchromatic, enlarged nuclei, and conspicuous nucleoli [68].	Gray tumor in the renal medulla that is poorly lobulated, firm, rubbery, and well-defined. Necrosis and hemorrhage are frequent [68].
	Unclassified renal cancer	Histologic characteristics that either share traits with more than one subtype or do not resemble any recognized subtype [69].	Large involvement of kidney and frequent necrosis [69].
2. Urothelial carcinoma		Squamous and glandular variants were the most common. The glandular variant is similar to enteric carcinoma and is gland-like or small tubular spaces in typical urothelial carcinoma. The squamous form can be distinguished by distinct intracellular keratin, intracellular bridges, or keratin pearl characteristics [40].	Squamous variant is usually large exophytic bulky tumor; some cases are predominantly flat with ulcerating/infiltrating appearance [70].
3. Wilms' tumor (nephroblastoma)		Epithelial, stromal, and blastemal components are arranged in a triphasic manner (Figure 1). Numerous tumor appearances are caused by the vast variations in these components' quantities, lines, and levels of differentiation [71].	A solid, pale grey to slightly pink or yellow-grey, soft-consistency tumor is typically present. Some tumors are clearly cystic, thus it's important to carefully check for the existence of solid foci [71].
4. Renal sarcoma		Plump ovoid cells are grouped in broad trabeculae or nests and divided by regularly spaced and arborizing fibrovascular septa. These septa, which have narrow capillaries or bands of fiber collagen that are abundantly thickened, are frequently described as having a "chicken-wire" appearance. The cord cells have uniform round to oval nuclei with fine chromatin, inconspicuous nucleoli, and transparent cytoplasm [61].	Appears as a fleshy cut surface that ranges in color from white to pale tan-grey and distorts most of the nearby healthy kidneys. The renal medulla might seem to be the mass center. Some tumors have cut surfaces that range from soft, mucoid to cystic and contain bleeding or necrosis foci [61].

Diagnostic imaging used in renal masses

Renal cancer is detected incidentally in most cases. A simple cyst is the most common finding, followed by complex cysts and solid masses. Magnetic resonance imaging (MRI), computed tomography (CT), contrast-enhanced ultrasound (CEUS), and ultrasound (US) are the most frequent imaging used for differentiating between malignant and benign masses and determining proper management [72].

The features of renal masses (except the angiomyolipoma containing fat and a typical simple cyst) require doing an MRI or CT scan after giving the contrast agents. There is no pre-agreed protocol regarding how to use MRI or CT for kidney masses, or characterization. However, a venous phase, a corticomedullary phase, and an unenhanced phase are substantial to identify the enhancement and to evaluate other characteristics including the type of lesion (infiltrative or expansive), the similarity of the enhancement (heterogenous or homogenous), and the vascularity compared to other parenchyma of the kidney (hypo-, iso-, hyper-enhancement) [72,73].

According to the most suitable imaging to characterize the masses of the kidney, the diagnostic capability of MRI study is as same as CT study concerning the existence and type of enhancement. However, almost all recommendations prefer using CT scans because of their quality with no artifacts, better resolution, lower cost, higher availability, and keep using MRI for challenging cases [74]. On the other hand, there are some advantages (i.e., the additional information given from the different sequences and the lack of ionizing radiation) that make MRI a better choice. Furthermore, the characteristics of the patients, possible contraindications, the complementary techniques, and the experience of the center affect the choice of the best imaging technique [75].

With the constant progress in diagnostic imaging, novel techniques have become incorporated like software of lesion segmentation to define with higher accuracy, for example, the tumor enhancement’s degree [76], dual-energy CT to quantify iodine [77], MRI perfusion and diffusion techniques [78], and quantification of tumor heterogeneity by CT texture analysis [79]. More research is needed to determine whether these tools are reliable and accurate enough to be used as part of the imaging approach used to evaluate the masses of the kidney [72].

Immunotherapy

One form of treatment for cancer is immunotherapy. It boosts the immune system and assists the body in locating and eliminating cancer cells by utilizing substances made in the body or a laboratory. Numerous types of cancer can be treated with immunotherapy. It can be used on its own or in conjunction with other cancer treatments like chemotherapy [80].

The finding that spontaneous regression sometimes happens in patients with metastatic renal cancer after surgical removal of the primary tumor has shown for the first time that renal cancer may be a suitable target for immunotherapy. Historically, immunotherapy in the form of immunostimulatory chemicals known as cytokines was a common first-line treatment for advanced renal cancer. They are now only used for cancers that don’t respond to targeted therapies because of serious side effects. Interleukin-2 and interferon-alpha help shrink kidney cancer in about 10-20% of patients and produce durable remission in a small percentage of these patients. In the treatment of advanced or metastatic kidney cancer, several recent immunotherapies, particularly checkpoint inhibitors have emerged as essential components [80].

Immune checkpoint inhibitors and cytokines are the two main types of immunotherapy that doctors use to treat kidney cancer. For our immune system to identify the potential threat and prevent it from destroying healthy cells, it uses proteins called checkpoints to trigger a response toward the potential threat. Protein checkpoints are sometimes used by cancerous kidney cells to prevent an immune response that could kill them. Immune checkpoint inhibitors stop cancer cells from getting rid of or avoiding the immune system's cells. The medication assists the immune system in identifying and attacking cancerous cells. In addition to that, the immune system is boosted by a small protein called cytokines. They can assist in the death of abnormal cells while keeping healthy cells alive for a longer period. Doctors use two types of cytokines made by humans to treat kidney cancer. Interleukin-2 and interferon-alpha are two examples [80]. Table 6 demonstrates various immunotherapeutic agents, their mechanism of action, and the types of renal cancer they are used against.

**Table 6:** Various immunotherapeutic agents, their mechanism of action, and the types of renal cancer they are used against.

Type	Name	Mechanism of action	Renal cancer
Immune checkpoint inhibitor (PD-1 inhibitor) [81]	Nivolumab	Blocks PD-1's interaction with its ligands by competing with PD-L1 for binding to PD-1	Renal medullary carcinoma
	Pembrolizumab		Renal cell carcinoma
Immune checkpoint Inhibitor (PDL-1 inhibitor) [82]	Atezolizumab	Blocks the binding of PDL-1 to PD-1 which allows T-cells to kill tumor cells	Urothelial carcinoma non-clear cell kidney cancer metastatic bladder cancer
	Avelumab		
	Durvalumab		
Immune checkpoint inhibitor (CTLA-4 inhibitor) [83]	Ipilimumab	Blocks the binding of CTLA-4 to another protein called B7 which increases the ability of T-cells to kill tumor cells	Metastatic clear cell cancer
Cytokine interleukin-2 [84]	Interleukin-2	Blocks the reproduction and spread of cancer cells. Also, it boosts the immune system	Metastatic renal cell carcinoma
Cytokine interferon alfa [84]	Interferon alfa	Stimulates t-cells and other immune system cells to kill cancer cells by sending chemical signals to attract them	Metastatic renal cell carcinoma

## Conclusions

Numerous forms of renal malignancies were covered in this review in terms of gross and histopathological appearance, diagnosis, and treatments. However, we gave more attention to RCC because it is the most common renal tumor. RCC causes approximately 14,000 mortalities and 63,000 new cases yearly in the United States.

Additionally, we focused on the genetic and acquired factors that may predict the prognosis of these tumors such as the E-cadherin gene for urothelial carcinoma and smoking, obesity, and asbestosis for renal sarcoma. Determining these factors is very important as they will provide insights into the natural history and biology of the tumors, as well as enable the physicians to choose the most appropriate treatment plan and optimize it regarding the patient's prognostic factors.
